# Molecular characteristics and chromatin texture features in acute promyelocytic leukemia

**DOI:** 10.1186/1746-1596-7-75

**Published:** 2012-06-28

**Authors:** Mariana R B De Mello, Dulcineia M Albuquerque, Fernanda Gonçalves Pereira-Cunha, Krizzia B Albanez, Katia B B Pagnano, Fernando F Costa, Konradin Metze, Irene Lorand-Metze

**Affiliations:** 1 Department of Internal Medicine, Faculty of Medical Sciences, State University of Campinas, Rua Tessalia Vieira de Camargo 126, 13083-887, Campinas, Brazil; 2 Hematology/Hemotherapy Center, State University of Campinas, Rua Carlos Chagas 480, 13083-878, Campinas, SP, Brazil; 3 Department of Pathology, Faculty of Medical Sciences, State University of Campinas, Rua Tessalia Vieira de Camargo 126, 13083-887, Campinas, Brazil

**Keywords:** Promyelocytic leukemia, Prognosis, FLT3-ITD, Chromatin texture

## Abstract

**Background:**

Acute promyelocytic leukemia is a cytogenetically well defined entity. Nevertheless, some features observed at diagnosis are related to a worse outcome of the patients.

**Methods:**

In a prospective study, we analyzed peripheral (PB) leukocyte count, immunophenotype, methylation status of CDKN2B, CDKN2A and TP73; FLT3 and NPM1 mutations besides nuclear chromatin texture characteristics of the **leukemic cells**. We also examined the relation of these features with patient’s outcome.

**Results:**

Among 19 cases, 4 had a microgranular morphology, 7 presented PB leukocytes >10x10^9^/l, 2 had FLT3-ITD and 3 had FLT3-TKD (all three presenting a methylated CDKN2B). NPM1 mutation was not observed. PB leukocyte count showed an inverse relation with standard deviation of gray levels, contrast, cluster prominence, and chromatin fractal dimension (FD). Cases with FLT3-ITD presented a microgranular morphology, PB leukocytosis and expression of HLA-DR, CD34 and CD11b. Concerning nuclear chromatin texture variables, these cases had a lower entropy, contrast, cluster prominence and FD, but higher local homogeneity, and R^2^45, in keeping with more homogeneously distributed chromatin. In the univariate Cox analysis, a higher leukocyte count, *FLT3*-ITD mutation, microgranular morphology, methylation of *CDKN2B*, besides a higher local homogeneity of nuclear chromatin, a lower chromatin entropy and FD were associated to a worse outcome. All these features lost significance when the cases were stratified for *FLT3*-ITD mutation. Methylation status of *CDNK2A* and *TP73* showed no relation to patient’s survival.

**Conclusion:**

in APL, patients with *FLT3*-ITD mutation show different clinical characteristics and have blasts with a more homogeneous chromatin texture. Texture analysis demonstrated that FLTD-ITD was accompanied not only by different cytoplasmic features, but also by a change in chromatin structure in routine cytologic preparations. Yet we were not able to detect chromatin changes by nuclear texture analysis of patients with the FTLD-TKD or methylation of specific genes.

## Background

Acute promyelocytic leukemia (APL) is a well characterized subtype of acute myeloid leukemia (AML) defined by a specific cytogenetic alteration of the tyrosine kinase 3 gene [1-5]. In Brazil, APL accounts for about 20% of the adult patients with *de novo* AML, which is a higher proportion than what is found in USA or Europe [1-7].

APL promyelocytes express regularly CD33, CD13 and CD117, and infrequently HLA-DR and CD34 antigen [8]. Although the disease is a cytogenetically clearly defined entity, several clinical and biological features have shown to be of prognostic importance, such as presence of the so-called variant (microgranular) morphology of the leukemic cells, high peripheral leukocyte counts at diagnosis or different RARα fusion partners [1-3]. A prognostic index based on peripheral leukocyte and platelet counts has been established by PETHEMA and GIMEMA Groups and validated also in brazilian patients [4]. Whereas in other AML subtypes, cytogenetic alterations and specific gene mutations are relevant for patients’ outcome, the prognostic relevance of additional karyotype abnormalities or gene mutations in APL patients are still controversial [1-3,6,7].

In APL, two mutations of the *fms*-related tyrosine kinase 3 gene ( *FLT3*) are more frequent: the internal tandem duplications ( *FLT3*-ITD) of the justamembrane region is found in 12-38% of the cases and the missense point mutations involving mainly the D835/I836 residues of the second tyrosine kinase domain ( *FLT3*-TKD) are observed in 2-20% of the patients [3] .

*FLT3*-ITD has been associated with high peripheral leukocyte counts at diagnosis and a lower disease-free survival [2,3,9,10]. The association of the *FLT3*-TKD mutation with the outcome of APL patients has been reported in only few studies, but with controversial results [3,7].

Epigenetic mechanisms play an important role in the pathogenesis of acute leukemias [11-19]. They are important for DNA stability as well as for the activation of several pathways of cell cycle regulation and apoptosis. Recent studies indicate that DNA methylation status and post-translational modifications of histones may be at least as important as gene mutations and deletions for the activation of oncogenes and silencing of tumor suppressor genes [12-14]. Methylation of specific genes involved in cell cycle control such as *CDKN2B* ( *p15*), *TP73* (p73) and *ESR1* ( *ER*) have been studied in AML and MDS [13,15,19]. Hypermethylation of *CDKN2B* has been associated with a poorer prognosis of the patients. There are only few investigations about the interaction between molecular alterations and DNA methylation profile in APL.

The interaction of genetic and epigenetic mechanisms leads to chromatin remodelling which may be measured in an objetive way by analysis of the nuclear chromatin texture in routinely stained slides. It has been demonstrated that in Giemsa-stained cells, the deeply stained heterochromatin domains correspond to the methyl-rich regions of CpG islands [20]. Therefore, the chromatin methylation pattern may be evaluated by computer-assisted analysis of the nuclear texture in cytological preparations. This principle has been applied to routine histological and cytological material of several solid tumors and hematologic neoplasias including AML, disclosing the prognostic importance of a variety of features of quantitative analysis of the nuclear chromatin pattern [16-19,21-29].

Special attention has always been drawn to cytoplasmic features of the APL blasts. To our knowledge, however, a nuclear texture analysis has never been performed in this type of AML. Thus, the aim of our study was to examine the relation among clinical and molecular features, more precisely, the relation between alterations in the *FLT3* gene, methylation of specific genes, nuclear chromatin texture characteristics and outcome in APL patients.

## Methods

### Patients

**Figure 1:**
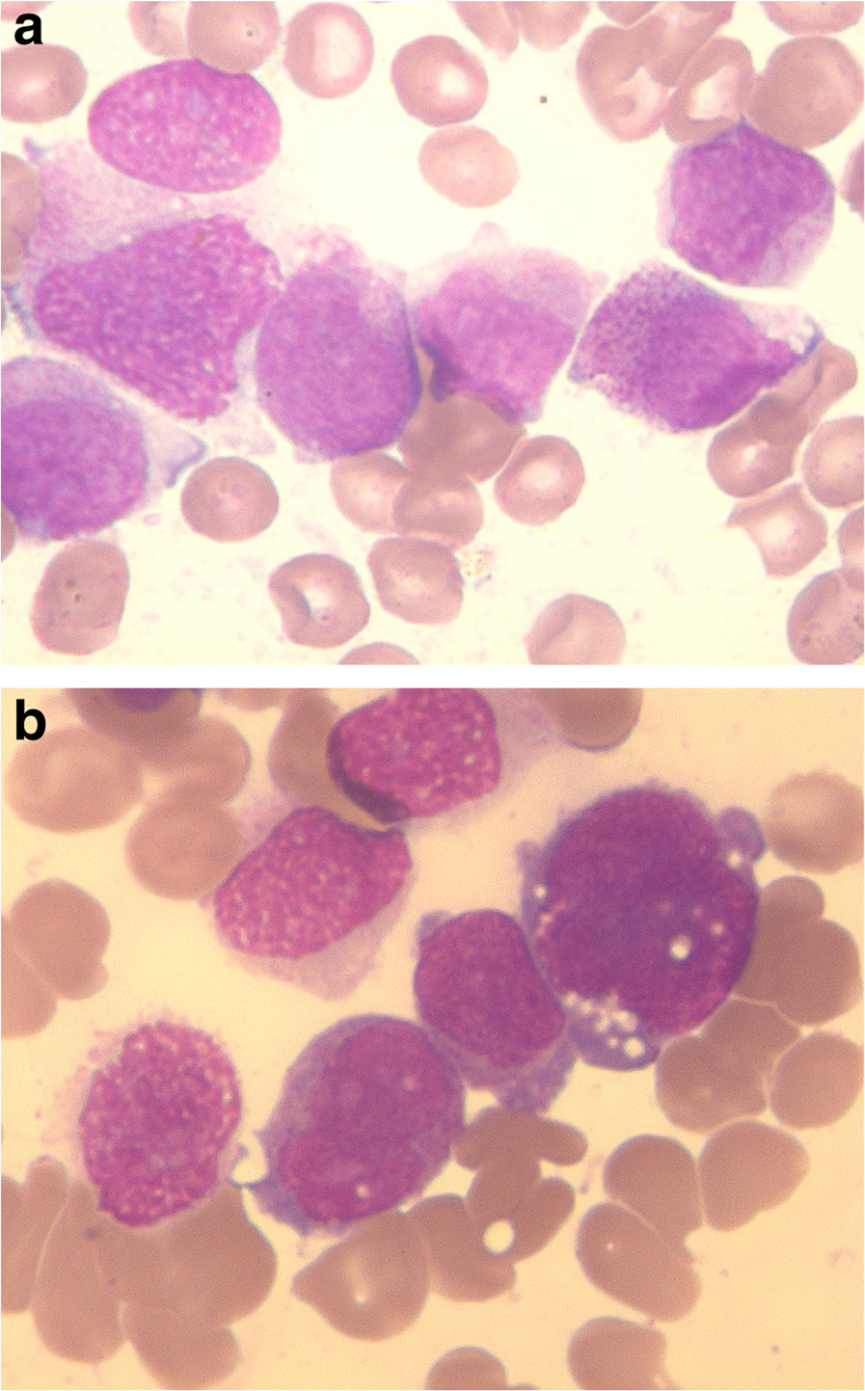
**Bone marrow smears of cases of APL.A** - classical morphology: the leukemic cells present a folded nucleus and a broad and hypergranular cytoplasm. Several cells present Auer rods (upper left and lower middle). **B** – variant morphology: the neoplastic cells present an oval or bilobated nucleus and few small granula in the less abundant cytoplasm. May-Grünwald-Giemsa. x1000..

The study included all consecutive new cases of *de novo* APL diagnosed at the Hematology and Hemotherapy Center of Campinas between 2007 and 2009. Peripheral blood (PB) counts, bone marrow (BM) examination, cytogenetics, immunophenotyping, texture analysis of nuclear chromatin, methylation of *CDKN2BCDKN2A* and *TP73* genes as well as mutations in *FLT3* were performed at diagnosis. According to morphology, cases were divided into those with the classical, hypergranular morphology (Figure 1A) and cases that showed predominantly a bilobated nuclear shape (Figure 1B) and few small granula in a less abundant cytoplasm (microgranular or variant morphology) [1].

All the patients were treated by the modified AIDA protocol [30,31]. Overall survival (OS) of the patients was calculated from the date of diagnosis to the date of death or last follow-up.

This study was approved by the Ethics Committee of Faculty of Medical Sciences of the University of Campinas (proc nr 389/2007).

### Immunophenotyping

A two-step tree color platform as described by Pereira et al [32] was used. The screening panel comprised three antibody combinations: CD3/CD19/CD45; CD7/CD13/CD45 and HLA-DR/CD33/CD45. If leukemic blasts expressed CD13 and/or CD33, the study was complemented with the combinations CD11b/CD14/CD45; CD15/CD34/CD45 and cMPO/CD117/CD45. For each sample at least 10,000 events were acquired on a FACs Calibur^TM^ equipment (Becton-Dickinson, San Jose – California -USA) with the Cell-Quest^TM^ (BD) software. Quantitative analysis was performed using the Paint-a-Gate^TM^ software (BD).

### Image analysis

Bone marrow slides at diagnosis, stained with May-Grünwald-Giemsa were retrieved from the files. Nuclear chromatin texture analysis was performed on at least 100 randomly choosen, non-overlapping tumor nuclei per patient using the Leica DC 500 system (ocular lens 10x and objective 100x, oil immersion). Neoplastic cells were acquired in 24-bit color bitmap format (12 megapixels per image) The nuclei were interactively segmented and then converted to 8 bit gray scale with levels of luminance ranging between 0 and 255.

We analyzed features of geometric morphometry (nuclear area, form factor 8, gray level and its standard deviation) and variables of the gray level co-occurrence matrix (entropy, contrast, local homogeneity and cluster prominence) [28,29]. We also determined the fractal dimension (FD) of the nuclear chromatin according to the Minkowski-Bouligand method extended to pseudo-3D images [25-28]. We performed a pseudo-3D transformation of the images, considering the x and y coordinates as the position of the pixel and the z axis was its grey level [25]. The FD of this surface was calculated using an in-house developed software. The goodness of fit of the FD (the adaptation of the real FD to the ideal one) was also determined, using a linear regression. The point distribution of the slope was rotated to 45 degrees as previously described [25]. The coefficient of the regression between the real and the estimated values was calculated. The R^2^ value obtained is an estimate of the “quality of fractality”. An ideal fractal has a R^2^ = 1.0. The real fractals have R^2^ values <1.0 [25,27].

### Methylation studies

For methylation studies, DNA samples were treated with sodium bisulfite [33]. Reactions for *CDKN2A* and *TP73* genes were performed by methylation specific PCR (MSP), and combined bisulfate restriction analysis (COBRA) was used to study the methylation status of *CDKN2B* gene. Primer sequences and temperature anneling are shown in Table 1. After amplification, the PCR products were digested with specific enzymes (Table 1) and the products were separated on 3% agarose gel stained with ethidium bromide.

**Table 1:** Sequences of primers and conditions used bisulfite PCR

***Primers***	**Sequences**	**Temperature (°C)**	**Enzyme**
*CDKN2B*	GGAGTTTAAGGGGGTGGG	58	*BstUI*
	CCTAAATTACTTCTAAAAAAAAAC		
*CDKN2A* U	TTATTAAGAGGGTGGGGTGGATTGT	60	-
	CAACCCCAAACCACAACCATAA		
*CDKN2A* M	TTATTAGAGGGTGGGGCGGATCGC	60	-
	AGGGGATGTAGTGAAATTGGGGTTT		
*TP73* U	AGGGGATGTAGTGAAATTGGGGTTT	60	-
	ATCACAACCCCAAACATCAACATCCA		
*TP73* M	GGACGTAGCGAAATCGGGGTTC	60	-
	ACCCCGAACATCGACGTCCG		

### Molecular study

#### Ouvir

Ler foneticamente

Dicionário - Ver dicionário detalhado

 1. substantivo

 2. temperature

The genotyping technique was used to study mutations in *FLT3*-ITD. MegaBACE 1000 equipment (GE Healthcare - Amersham) was used and analysis was made with the Fragment Profiler v1.2 software. In brief, 0.5 μl DNA was amplified in a volume of 50 μl containing 120 mm *FLT3* sense primer labeled with HEX fluorophore (Table 2) and 120 mm *FLT3* antisense primer unmarked; 10X buffer, 3 mM MgCl2, 0.2 mM dNTP's, 0.05 U Taq polymerase and water. It consisted of an initial denaturation step at 94°C for 10 minutes followed by 35 cycles at 94°C for 30 seconds, 56°C for 1 minute, and 72°C for 2 minutes and a final extension step at 72°C for 7 minutes. PCR products were diluted 40x in water. Then, 2 μl of each dilution was distributed in plates with 96 wells previously prepared with a mixture containing 7.75 μl Genotyping Loading Solution (0.1% Tween 20 in H_2_O) + 0.25 μl of MegaBACE ET550-R Size Standards, making a final volume of 10 μl. Samples were denatured at 95°C for 5 minutes before being placed on the equipment.

**Table 2:** **Sequence of p* rimers *and enzymes used in* FLT3 *reactions**

***Primers***	**Sequence**	**Enzyme**
*FLT3*-ITD	GCAATTTAGGTATGAAAGCCAGC	-
	CTTTCAGCATTTTGACGGCAACC	
*FLT3*-TKD	CCGCCAGGAACGTGCTTG	*EcoRV*
	GCAGCCTCACATTGCCCC	

PCR for the detection of *FLT3*-TKD mutation was performed using 0.025U Taq DNA polymerase, 200 nM of each primer (Table 2), 0.2 mM dNTP's, 2 mM MgCl_2_, 10x buffer and water. Cycling conditions: 94°C for 5 for minutes, 40 cycles of 94°C for for 1 minute, 55°C for 40 seconds, 72°C for 30 seconds and final extension at 72°C for 5 minutes. After amplification PCR products were digested with enzyme *Eco*RV and products were separated by electrophoresis on 3% agarose gel stained with ethidium bromide.

### Statistical analysis

The differences between groups were examined by the Mann–Whitney test. Correlations were examined by the Spearman rank order correlation. The differences were considered significant when *p* < 0.05. The relations between clinical, phenotypic, morphometric and molecular features with OS of the patients were analysed using the Cox proportional hazard regression. Because of the small number of events we did not perform a multivariate analysis. Instead, we compared the univariate Cox-regressions for each of the variables studied before and after stratification for the presence of *FLT3-ITD*. Winstat 3.1. and SPSS 15 programs were used. The exact confidence intervals were calculated with the free graph pad software (t http://www.graphpad.com/quickcalcs/ConfInterval1.cfm)

## Results

We detected 19 cases of APL in a cohort of 106 consecutive cases of *de novo* AMLs (17.9%), diagnosed at our Institution during the period of the study. Among them, 10 patients were diagnosed by classical cytogenetics: 8 cases had only t(15;17); 1 had t(4;6), t (15;17); 1 had del(7), t(15;17). Six cases were diagnosed only by molecular diagnostics ( *PML*/ *RARα*) and in 3 cases the diagnosis was made only by morphologic criteria, since metaphases could not be achieved for cytogenetic examination.

Clinical data and PB counts are shown in Table 3. Patients had a median age of 44 years. PB leukocyte counts were >10x10^9^/l in 7 cases. Concerning the immunophenotypic features, 2 cases showed an expression of HLA-DR (mean fluorescence intensity – MFI - of 100 and 141) and four had a dim expression of CD34 (mean MFI of 47). Four cases presented microgranular morphology; all of them presented a high PB leukocyte count (Table 4). Concerning nuclear texture features, these cases had higher local homogeneity, contrast and R^2^ values, but a lower standard deviation of gray levels, entropy, cluster prominence, and fractal dimension.

**Table 3:** Clinical and hematological data of the patients

**Age (years)**	**44 (20–82)**
Male/Female	9/10
Hemoglobin (g/dl)	8.8 (3.0-14.1)
Leukocytes (x10^9^/l)	5.0 (0.69-160.0)
Platelets (x10^9^/l)	18.5 (5.0-85.0)

**Table 4:** Peripheral leukocyte counts and morphometric features of patients with APL according to the morphological type (median and range)

	**Variant (microgranular)**	**classical**	***P***
Leukocyte count x10^9^/l	63.0 (24.0 – 160.0)	1.75 (0.69 – 68.0)	0.007
Nuclear area (μ^2^)	86.5 (83.4 – 115.3)	113.4 (87.1 – 115.3)	0.15
Form factor	1.11 (1.02 - 1.12)	0.97 (0.80 - 1.12)	0.03
**Mean gray level**	**109.7 (106 – 110)**	**129.7 (102 – 158)**	**0.012**
SD gray level *	6.4 (6.2 - 7.6)	8.7 (7.3 - 10.4)	0.018
Local homogeneity	0.59 (0.58 - 0.61)	0.55 (0.53 - 0.58)	0.008
Entropy	7.01 (6.89 – 7.32)	7.76 (7.29 – 8.21)	0.01
Contrast	2.88 (2.29 - 3.15)	2.59 (2.29 - 2.89)	0.02
Cluster Prominence (x10^5^)	2.20 (1.97 - 4.48)	5.29 (2.26 – 11.94)	0.05
FD Minkowski	2.09 (2.08 - 2.10)	2.11 (2.10 - 2.13)	0.01
R^2^	0.99910 (0.99900 - 0.99940)	0.99885 (0.99802 - 0.99910)	0.02

* Standard deviation of gray level.

Peripheral leukocyte count at diagnosis had no relation with phenotypic features, but showed an inverse correlation with standard deviation of gray levels (r = −0.49; p = 0.03), contrast (r = −0.48; p = 0.03), cluster prominence (r = −0.57; p = 0.01) and fractal dimension (r = −0.46; p = 0.04).

*FLT3*-TKD mutation was found in 3 cases (15.8%; 95% confidence interval from 0.0478 to 0.384) and *FLT3*-ITD was present in 2 cases (10.5%; 95% confidence interval 0.013 - 0.331). All patients presenting *FLT3*-TKD had also a methylated *CDKN2B* gene. This gene was methylated in only one patient with no abnormalities in *FLT3*. *CDKN2A* was methylated in 21.0% (95% confidence interval 0.0605 - 0.4557) and *TP73* in 10.5% of the patients (95% confidence interval 0.0130 - 0.3314).

The leukemic cells of patients with *FLT3*-ITD expressed HLA-DR, CD34 and CD11b (Table 5). They also had higher PB leukocyte counts. Both presented a variant morphology. Concerning nuclear texture features (Table 6), these patients presented a lower standard deviation of grey levels, entropy, contrast, cluster prominence and the fractal dimension (FD), but higher values for local homogeneity, and the goodness-of-fit of the FD.

**Table 5:** **Hematological and phenotypic features of the patients according to presence of*FLT3*-ITD**

	***FLT3*-ITD +**	***FLT3*-ITD -**	***p***
Hemoglobin (g/dl)	8.8 (8.0-9.7)	8.7 (3.0-14.1)	0.78
Leukocytes (x10^9^/l)	63.0 (61.0-65.0)	2.0 (0.69-160.0)	0.09
Platelets (x10^9^/l)	27.0 (20.0-34.0)	14.5 (5.0-85.0)	0.32
MFI HLA-DR	121.0 (100.8-141.2)	0.0 (0.0-25.9)	0.0004
MFI CD11b	41.1 (39.8-42.5)	0.0 (0.0-20.1)	0.01
MFI CD34	42.9 (35.4-50.4)	0.0 (0.0-63.5)	0.009

**Table 6:** **Morphometric features of patients with APL according to*FLT3*-ITD**

	***FLT3*-ITD +**	***FLT3*-ITD -**	***p***
Leukocyte count x10^9^/l	63.0 (61.0 – 65.0)	2.0 (0.7 – 160.0)	0.04
Nuclear area (μ^2^)	100.9 (86.6-115.3)	105.4 (83.4-126.9)	0.63
**Form factor**	**1.07 (1.02 - 1.12)**	**0.97 (0.80 - 1.12)**	**0.07**
**Mean gray level**	**108.2 (106 – 109)**	**129.7 (102 – 158)**	**0.028**
Standard deviation of gray level	6.3 (6.2-6.5)	8.7 (7.4-10.4)	0.026
Local homogeneity	0.60 (0.59-0.61)	0.55 (0.53-0.58)	0.026
Entropy	6.95 (6.90-7.01)	7.74 (7.29-8.21)	0.026
Contrast	2.59 (2.29-2.89)	3.69 (2.87-4.83)	0.04
Cluster Prominence (x10^5^)	2.09 (1.97-2.2.20)	5.04 (2.46-11.94)	0.026
FD Minkowski	2.10 (2.09-2.10)	2.12 (2.10-2.14)	0.05
R^2^	0.99925 (0.99910-0.99940)	0.99841 (0.99802-0.99910)	0.03

During the period of the study (median 23 months; 1 – 41 months) 5 patients had died, four of them within the first month of diagnosis. All these four had a high leukocyte count, and presented microgranular morphology or had *FLT3*-ITD. Using the Cox model, in the univariate analysis a higher leukocyte count (B = +0.032; *p* = 0.038), presence of *FLT3*-ITD mutation (B = +2.55; *p* = 0.01), a microgranular morphology (B = +2.39; *p* = 0.01), methylation of *CDKN2B* gene (B = +1.70; *p* = 0.066), expression of HLA-DR (B = +0.017; *p* = 0.025) and CD34 (B = +0.028; *p* = 0.077) as well as a higher local homogeneity of nuclear chromatin of the leukemic cells (B = +54.24; p = 0.035) were associated with a shorter overall survival. However, higher values of standard deviation of gray level (B = −1.28; p = 0.025), of entropy (B = −3.63; p = 0.031) and chromatin fractal dimension (B = −85.7; p = 0.036) were associated to a longer survival. All these values lost significance when the univariate Cox regressions were stratified by presence or absence of the *FLT3*-ITD mutation. Methylation status of *CDNK2A* and *TP73* showed no relation with the survival of the patients.

## Discussion

The outcome of patients with APL has improved over the last 2 decades as a consequence of a precise molecular diagnosis, better supportive care as well as the use of *trans*-retinoic acid (ATRA) and arsenic trioxide in chemotherapy protocols [30,34]. Nevertheless, 20% of the patients still die during the first month of treatment, mainly due to bleeding. So, it would be interesting to detect the clinical and laboratory features that could predict a shorter overall as well as disease-free survival. Peripheral leukocyte counts above 10x10^9^/l and a microgranular morphology have been recognized as such variables [2-5]. Additional chromosomal abnormalities, however, do not seem to influence the outcome of the patients [6,7]. Therefore, it would be interesting to study the relation of these features with molecular characetristics of this disease. Nuclear texture features of neoplastic cells have shown to be morphological correlates of chromatin remodelling with genetic and epigenetic alterations in acute leukemias [12-17], and have shown to be related to patients’ survival. Recently, computerized image analysis has been used to examine chromatin remodeling in several hematological neoplasms and texture variables of the nuclear architecture have shown to be independent prognostic factors in acute leukemias [16-18,23,26,27,29].

In the present study, in a rather small cohort of patients, diagnosed and treated according to the international standard of care, we could observe a mortality rate of 21% during the first month of treatment which was similar to that observed in other countries [4,30,34]. We could also confirm the prognostic value of peripheral leukocyte counts and microgranular morphology.

More recently, several investigations have focused on the impact of mutations of the FLT3 gene in APL [2,3,5,7,9-11,35] that has been a well recognized adverse prognostic factor in AML with a normal karyotype. In APL, the finding of FLT3-ITD has been associated with increased peripheral leukocyte counts, higher early mortality and more frequent recurrences. The presence of FLT3-ITD in APL is also associated with a different expression of 147 genes involved in cytoskeleton organization, cell proliferation and migration, adhesion, as well as the coagulation and inflamation pathways [36]. Thus, it is linked to a more aggressive clinical behaviour with higher peripheral leukocytosis and a more pronounced coagulopathy, provoking a higher rate of early death, mainly due to bleeding. The higher leukocytosis is also indicative of a more pronounced proliferative activity. Our study also corroborated these findings. FLT3-ITD was a significant adverse prognostic factor for survival in the univariate Cox-regression. Furthermore, other variables, such as peripheral leukocyte count, microgranular morphology and texture features of nuclear chromatin were significant prognostic factors only when examined isolately, but lost their importance when the regressions were stratified for the presence of FLT3-ITD. From these findings we may hypothesize that the presence of the FLT3-ITD mutation might be more important for a dismal outcome than leukocytosis alone or a microgranular morphology. Additional studies with larger number of patients, however, are necessary to clarify this question.

Neither the FLT3-TKD mutation nor the methylation of *CDNK2B*, were unfavourable prognostic features in our investigation. Yet, a possible role of *CDNK2B* for the outcome of patients with other types of acute leukemias has been postulated [37]. Bit this could not be confirmed for APL in the present study, perhaps due to the reduced test power caused by the small cohort of patients.

Recently, the importance of epigenetic changes for normal hemopoietic maturation as well as in the pathogenesis of AML has been emphasized [14,17-19]. Hypermethylation of CpG islands within the promoter regions together with deacetylation of certain histones is an important mechanism of gene silencing in hematologic neoplasias. Several studies have shown that APL is generally associated with a specific methylation pattern, but some genes, such as CDKN2B, may be methylated isolately in only a part of the patients [15,19]. Patients with the FLT3-ITD mutation show a gene expression profile where various genes relevant for cytoskeleton organization, cell adhesion and migration, proliferation and coagulation pathways are expressed [36].

MGG staining of cytologic smears permits to evaluate the topographic localization of methylated regions in the nucleus, since deeply Giemsa-stained compact heterochromatin domains are co-localized with methyl-rich CPG islands [20]. Computer-assisted image analysis is able to document discrete changes in the chromatin structure, which may not be visible for the trained observer [38-43]. Discrete morphologic alterations of the nucleus and its substructures accompany functional and molecular changes of the cell. This is also true for benign and malignant hemopoesis [44-48].

High order chromatin architecture alterations may parallel chromosomal alterations in cancer [44,45]. Moreover, disrcrete morphologic changes of the chromatin texture may be equivalent to alterations of the methylation pattern and therefore of the gene expression. Probably for these reasons computerized texture analysis of chromatin has shown to be helpful for diagnosis as well as for prognosis of several neoplasias including AML [16-18,23-28,38-43]. In particular, a fractal model of nuclear chromatin has been associated with cellular activity, the organization of nuclear chromatin and the surrounding nucleoplasmic space, especially the distribution of heterocromatin, and with prognosis [16,25-29,46].

In our study several variables of geometric morphometry, the gray-level co-ocurrence matrix and also R^2^ showed different values in leukemic cells of cases with and without the FLT3-ITD mutation. In patients presenting FLT3-ITD, cells had a smoother and more homogeneously distributed chromatin which would more likely correspond to profound alterations of the epigenome. The presence of FLT3-ITD has shown to be associated with a higher proliferative activity and a more undifferentiated phenotype [36]. This is in keeping with the different chromatin texture features found in our patients as well as the higher PB leukocytosis.

Several clinical, phenotypical and nuclear chromatin texture features were associated to the survival of our patients. However, when stratified for the presence of FLT3-ITD, all of them lost their prognostic value. In that way, our explorative study indicated that this mutation is a very important prognostic factor in APL. This is in keeping with the concept of a previous study that APL with FLT3-ITD mutation represents a distinct subtype of APL with a worse prognosis [36].

In conclusion, our work underlines the necessity of the detection of the FLT3-ITD mutation in APL, that constitutes a separate entity of a worse prognosis and where new treatment strategies are necessary in order to decrease early mortality in an otherwise highly curable form of acute leukemia. Texture analysis demonstrated that the FLTD-ITD alterations were accompanied not only by different cytoplasmic features, but also by a change in the chromatin structure in routine cytologic preparations. Yet we were not able to detect chromatin changes by nuclear texture analysis of patients with the FLTD-TKD mutation or methylation of specific cell genes.

## Competing interests

The authors have no conflicts of interests to declare.

## Author’s contributions

MRBM collected the clinical data, contributed to the study design, performed all the molecular studies and wrote the manuscript draft. The results are a part of her PhD thesis, supervised by Irene Lorand-Metze (Postgraduate Course in Medical Pathophysiology, University of Campinas). DMA trained and supervised MRBM in the molecular techniques, and helped in the data interpretation. FGPC performed the diagnostic immunophenotyping of the patients and helped in the interpretantion of the data. KBA performed the acquisition of all nuclear images and made their segmentation. KBBP made the diagnostic molecular study and treated the patients. FFC supervised the molecular studies. KM contributed to essential parts of the the study design, performed the statistical analysis, interprettion of the results, wrote essential parts of the manuscript and made a critical review of the final version. ILM: conception and study design. Supervision of the collection of the data, microscopic analysis, interpretation of the data, manuscript draft and final approval. All authors have read and approved the manuscript.
